# Perspectives of individuals with chronic spinal cord injury following novel balance training involving functional electrical stimulation with visual feedback: a qualitative exploratory study

**DOI:** 10.1186/s12984-021-00861-z

**Published:** 2021-04-01

**Authors:** David J. Houston, Janelle Unger, Jae W. Lee, Kei Masani, Kristin E. Musselman

**Affiliations:** 1 KITE Toronto Rehabilitation Institute-University Health Network, Toronto, Ontario Canada; 2 Rehabilitation Sciences Institute, Temerty Faculty of Medicine, University of Toronto, Toronto, Ontario Canada; 3 Institute of Biomedical Engineering, Faculty of Applied Science & Engineering, University of Toronto, Toronto, Ontario Canada; 4 Department of Physical Therapy, Temerty Faculty of Medicine, University of Toronto, Toronto, Ontario Canada

**Keywords:** Visual feedback, Balance training, Functional electrical stimulation, Spinal cord injury, Neurorehabilitation

## Abstract

**Background:**

Individuals with an incomplete spinal cord injury (iSCI) are highly susceptible to falls during periods of walking or standing. We recently reported the findings of a novel intervention combining functional electrical stimulation with visual feedback balance training (FES + VFBT) on standing balance abilities among five individuals with motor iSCI. However, the previous publication did not report the perceived impact of the intervention on the participants’ lives. In this report, the experiences of these five individuals with incomplete spinal cord injury (iSCI) who had recently completed the four-week balance training program are described.

**Methods:**

Five individuals with a motor iSCI took part in this study. Each individual was at least 12 months post-injury, capable of unassisted standing for 60 s and had a Berg Balance Scale Score < 46. Participants completed twelve sessions of a novel balance intervention combining closed-loop functional electrical stimulation with visual feedback balance training (FES + VFBT). Participants received visual feedback regarding their centre of pressure position as they completed balance-training exercises while FES was applied to the ankle plantarflexors and dorsiflexors bilaterally. Semi-structured interviews were conducted after completion of the balance training intervention and eight-weeks post-training to understand participant’s experiences. Categories and themes were derived from the transcripts using conventional content analysis.

**Results:**

Three themes were identified from the collected transcripts: (1) Perceived benefits across International Classification of Functioning, Disability and Health levels; (2) Change in perceived fall risk and confidence; (3) Motivation to keep going.

**Conclusions:**

Participation in the FES + VFBT program resulted in perceived benefits that led to meaningful improvements in activities of daily living. Following completion of the training, individuals felt they still had the capacity to improve. Individuals felt they had increased their balance confidence, while a few participants also reported a decrease in their risk of falling. The inclusion of qualitative inquiry allows for the evaluation of the meaningfulness of an intervention and its perceived impact on the lives of the participants.

*Trial registration:* NCT04262414 (retrospectively registered February 10, 2020)

## Background

Damage to the spinal cord disrupts the communication between the motor cortex and the spinal cord resulting in sensorimotor impairments below the level of injury. Individuals with motor incomplete spinal cord injury (iSCI) often regain some degree of standing and walking abilities [1], due to residual motor functioning preserved below the level of injury. The ability to stand independently is an important contributor to an improved quality of life among individuals with SCI. It has been reported that individuals with SCI who engage in prolonged periods of standing experience psychosocial benefits, such as increased feelings of independence and self-efficacy, as well as physical benefits, such as improved circulation, and bladder and bowel functioning [2].

Independent standing, however, is often difficult for individuals living with motor iSCI to achieve. Their control of standing balance is compromised due to the sensorimotor deficits associated with spinal cord damage leading to an increased reliance on visual inputs [3, 4] and an inability to appropriately modulate movements relative to task demands [5, 6]. As a result, individuals with iSCI are highly susceptible to experience a fall. Falls primarily occur during periods of walking or standing within the home [7] and are of significant concern due to the likelihood of injury or hospitalization [8]. In instances where an injury is not sustained, the occurrence of a fall is still sufficient to influence behavioural changes intended to restrict mobility on account of a learned fear of falling [9]. Among individuals with iSCI, 50% report a fear of falling [10]. Individuals with a fear of falling have been shown to exhibit reduced postural control [11] and increased fall risk [12].

Individuals with iSCI exhibit lower levels of confidence in their ability to maintain balance while performing specific daily activities than age-and sex-matched able-bodied controls, as assessed by the Activities-specific Balance Confidence (ABC) Scale [10]. Significant correlation between ABC Scale score and anterior–posterior (AP) centre-of-pressure (COP) velocity suggests a link between balance confidence and postural steadiness [10]. Means to improve postural steadiness may result in increased balance confidence, which may encourage individuals to engage in activities, hence increasing their functioning and independence.

We recently reported the findings of a 12-session balance training intervention combining functional electrical stimulation with visual feedback balance training (FES + VFBT) on standing balance abilities among five individuals with motor iSCI [13]. Participants received visual feedback regarding their COP position as they completed four balance-training exercises while FES was applied to the ankle plantarflexors and dorsiflexors. We demonstrated positive effects of FES + VFBT on standing balance ability as assessed by the Berg Balance Scale (BBS), the Mini-Balance Evaluation Systems Test (mini-BESTest), and the limits of stability test.

The initial report, however, lacked insight into the perceived impact of the intervention on the participants’ lives [13]. Meaningfulness, or whether the intervention outcome is important to the target population, is an important construct to assess early in the development of a new intervention [14]. Therefore, the purpose of this study was to understand the experiences of the five individuals who had completed the FES + VFBT intervention. More specifically, we aimed to understand how the intervention impacted their daily lives and their perceived risk of falling and balance confidence.

## Methods

Research ethics approval for this exploratory qualitative study was obtained from the University of Toronto and the University Health Network.

### Participants

A purposeful sample [15] of five individuals with a motor iSCI (i.e. American Spinal Injury Association Impairment Scale (AIS) rating of C or D) who previously took part in FES + VFBT [13] participated in this study (Table 1). These five participants were the same individuals whose balance performance and perceptions regarding user acceptability of the FES + VFBT intervention were reported in the previous publication [13]. Each individual was at least 12 months post-injury, capable of unassisted standing for 60 s, had a BBS Score < 46 prior to starting the FES + VFBT intervention, and had completed 12 sessions of the FES + VFBT intervention. The qualitative data collection described here was planned a priori and participants were not informed of their scores on the clinical and lab-based measures of balance [13] in advance of the interviews.

**Table 1:** FES + VFBT participant demographics

Participant pseudonym	Age category	Mechanism of injury	Level of injury	Time post-injury (months)	BBS (/56)	*AP-COP RMS (mm)	*ML-COP RMS (mm)
Charlie	65–69	NT	Thoracic	97–108	27	12.32 (2.04)	9.28 (1.45)
Sharon	65–69	NT	Cervical	49–60	31	NA	NA
Ruth	60–64	T	Cervical	13–24	42	5.90 (0.415)	10.48 (1.04)
Carol	60–64	NT	Cervical	25–36	48	7.57 (0.875)	8.79 (5.14)
Suzanne	55–59	NT	Thoracic	25–36	30	5.72 (1.10)	4.54 (0.825)

*NT* Non-Traumatic, *T* Traumatic, *BBS* Berg Balance Scale, *COP* centre-of-pressure, *RMS* root mean square, *AP* anterior–posterior, *ML* medial–lateral, *NA* not assessed due to fatigue

^*^COP data were collected during two 60 s trials of quiet standing with eyes open; values are reported as mean (SD). BBS scores and COP data collected at the post-training assessment

### FES + VFBT intervention

Participants completed 12 one-hour training sessions of FES + VFBT over four weeks. Four COP-based exercises, designed to encourage the participants to shift their COP in a multitude of directions, were performed at each session. As participants performed these movements, FES was applied bilaterally to the ankle plantarflexors and dorsiflexors to provide assistance as they attempted to move the location of their COP as instructed by the exercise. FES was regulated in a closed-loop manner where COP position was continually monitored to determine the amount of electrical current needed to assist the participant reach the desired target. Further information pertaining to the specifics of the training intervention can be found in Houston et al. [13].

### Data collection

Two semi-structured individual interviews were completed with each participant. One interview was completed immediately (i.e. 2–3 days) post-training and the second interview was completed eight-weeks post-training. Participants were interviewed at these two time points to examine whether the participants’ perceptions about the impact of FES + VFBT changed over time. At the eight-weeks post-training interview, participants were provided with the opportunity to review the transcript from their initial interview in order to clarify or add to any of their responses. Individual interviews were conducted following a semi-structured interview guide. The guide consisted of open-ended questions (Table 2) that encouraged participants’ to express their thoughts and experiences regarding the perceived impact of the FES + VFBT intervention. This interview guide was adapted from a guide previously used in another intervention study investigating participant perspectives [16]. Each interview was conducted in person (9 interviews) or over the phone (1 interview) by a researcher not involved in the delivery of the FES + VFBT intervention (JU or KEM). Interviews were audio-recorded and transcribed verbatim by a researcher (DJH) with all personal identifiers removed to protect the privacy of the participants.

**Table 2:** FES + VFBT semi-structured interview guide

We would like to hear about your experiences with FES for standing balance
What were you hoping to achieve by taking part in the balance training with FES?
Did you achieve these goals?
Were there any effects that you were not expecting?
Has your participation in the FES balance training impacted your life? How so?
Has your participation in the FES balance training affected your risk of falling? How so?
Has your participation in the FES balance training affected your balance confidence? How so?
Would you recommend balance training/walking training to another individual with an incomplete spinal cord injury? What advice would you give to someone who was about to begin the training program?

At the second interview, participants were instructed to reflect on the period of time following training completion when answering questions 2–4

Responses to questions were reviewed prior to the second interview and participants were asked to add or clarify, as they felt necessary

### Data analysis

Conventional content analysis [17], which involves a descriptive approach to qualitative data, was used to analyze the interview transcripts. Each transcript was read multiple times by two independent reviewers (DJH & JU). One researcher (DJH) was directly involved in administering the FES + VFBT program while the other (JU) was not. The immediate post-training transcripts were read and analyzed separately from the eight-week post-training transcripts. Quotes were highlighted from the text and placed in a table. Following steps from Erlingsson et al., [18] quotes were condensed in a manner preserving the core meaning and then assigned codes. Related codes were grouped together to form categories; interpreting the underlying meaning of each category identified themes. An additional reviewer (KEM), who was not directly involved in the delivery of FES + VFBT, reviewed the transcripts as well as the themes and associated categories. The primary reviewer (DJH) was a registered kinesiologist who has conducted quantitative research in non-SCI populations. The secondary reviewer (JU) was a physiotherapist with three years of experience in SCI rehabilitation and has engaged in both quantitative and qualitative research within this population. The tertiary reviewer (KEM) was a physiotherapist with 16 years of SCI rehabilitation experience who is well versed in both quantitative and qualitative research. To describe the perceived impact of the FES + VFBT intervention on the participants’ lives, the International Classification of Functioning, Disability and Health (ICF) framework was used to organize and describe the perceived benefits in terms of functioning and disability [19].

## Results

Five participants (1 male and 4 females, ages 55–69 years old) completed two interviews each (range: 17–52 min). Age, injury mechanism, neurological level of injury, time post-injury and post-training balance measurements for each participant are reported in Table 1. Three themes were identified through the analysis of interviews. Each theme was comprised of several categories with shorter supporting quotes integrated into the text, and longer quotes presented in Table 3, which have been linked to statements within the text. The integration of shorter quotes allows the findings to be presented in the participants’ voices, while the longer quotes may better represent their experiences [20]. The three main themes were: (1) Perceived benefits across ICF levels, (2) Change in perceived fall risk and confidence, and (3) Motivation to keep going. These themes were consistent across the interviews completed immediately post-training and the interviews completed eight-weeks post-training; hence the findings from the two interview time points are reported together. However, one difference was noted for the first theme (perceived benefits across ICF levels) when comparing these two time points. At the eight-week post-training interview, participants tended to describe activity-level and participation-level impact of the intervention on their lives, whereas they tended to describe impairment-level impact immediately post-training.

**Table 3:** Functional Electrical Stimulation with Visual Feedback Balance Training (FES + VFBT) Themes, Categories and Quotes

Quotes for Theme 1: Perceived benefits across ICF levels	
Category 1a Impact on impairment	[Q1] “…I honest to God believe it helped some kind of neural pathway…I honest to God believe that something happened between this muscle turning on and my brain because now it’s more active; I can control it to some extent…the stim actually activated that muscle where I might not have been able to do it just on my own…after having it stimulated for that amount of time…it’s for sure improved.” (Charlie, 8-Weeks Post-Training) [Q2] “…I had a visit with [my doctor] last week and she said, ‘Oh my God, your legs!’ And I mentioned, I mentioned this program…she said that they are getting stronger and stronger…I haven’t had spasms for a while. I don’t know if it is because of the stim…I still have, but not as bad as before… I told her about the neuropathic pain, this leg, and she said keep an eye on it. When I saw her, because she told me to write down how many times, she said ‘So, how many?’ I said, ‘One time, but for a few minutes and it was gone’…I feel happy.” (Carol, Post-Training) [Q3] “Just the increase of sensory, you know tactile, in my feet, and my skin and my muscles that weren’t…I wasn’t expecting that. I wasn’t expecting to have an increase in sensory. I was hoping that, you know, it would help my muscles and things, but that was a big thing.” (Charlie, Post-Training) [Q4] “Feeling of accomplishment, I think. Like I say, some of the stuff was minute, but it’s big. And you have the feeling…like to have my muscles come back and have the feeling…like a sense of feeling down there, sensory, that was pretty big.” (Charlie, Post-Training)
Category 1b Impact on activity	[Q5] “It’s nice that I do things around the house. I do more things now…I walk out to the backyard a lot, with a walker…over uneven ground, but I’m solid…I BBQ on my own now…I use a walker when I’m there, but I’m standing…I didn’t enjoy my backyard last year as much as I did this year.” (Charlie, 8-Weeks Post-Training) [Q6] “…it’s given me more confidence to get up and about around the apartment…in my home, I’m up and about more…taking stuff out of the washing machine and putting stuff in the dryer…yesterday I stood at the kitchen counter and peeled a mango. It doesn’t sound like much, but I stood there and I peeled it and I diced it and I put it into a container.” (Sharon, 8-Weeks Post-Training) [Q7] “The standing endurance, and the walking endurance. I mean I stood up the other day, now it was mostly on my right leg, but I was standing at my walker out on the balcony for, I would say, 25 min the other day.” (Sharon, 8-Weeks Post-Training) [Q8] “…I don’t use the chair anymore inside the home…only maybe once in a while, like very bad days; maybe then. But I avoid it and I am managing up to now. So I should say this [is] improvement, huge improvement for me…my mobility got [better], but once I get into the chair I don’t feel like to get up and do something…” (Ruth, 8-Weeks Post-Training)
Category 1c Impact on participation	[Q9] “…I actually think things are better, I can’t name an instance, but I feel better about my situation; I feel better about my legs. Like I say, just do more stuff and it’s not a problem. I’ve been to the beach twice…and I’m going golfing two weeks from now.” (Charlie, 8-Weeks Post-Training) [Q10] “I went to the mall and I went to the bank, I went to that food court area…with the walker…if I go out, the next day I relax. Then after that I go out.” (Suzanne, Post-Training) [Q11] “My friend has a van…big van. So of course I have to use the stool because it’s a high step…last Friday I put the stool, I have a folding stool, and immediately I told him to take the walker, put it in the trunk and he said, ‘Can you do it on your own?’ I said, ‘Of course I can do it on my own!’ You see, I was very confident. I’m not scared…he was surprised…I told him about this training session…those sessions helped me a lot.” (Carol, 8-Weeks Post-Training)
Category 1d Inter-related across ICF	[Q12] “I walk straighter because I know where my feet are…when I activate my toes now in my right foot …I know where it is so when I’m walking, I get easier feedback and I’ve found myself walking straighter.” (Charlie, Post-Training) [Q13] “Standing more in the kitchen…if I have to cook, chop, do the dishes…before like I used to stand, but the walker used to be behind me. Now I forget about the walker; well, I have a cane. I feel stronger, and I feel that my legs are stronger…” (Carol, Post-Training)
Quotes for Theme 2: Change in perceived fall risk & confidence	
Category 2a Risk of falling	[Q14] “Has it affected my risk? I don’t know. It’s minimized it more than ever. I’ve never really [been] afraid of falling, but I put myself in positions now where I don’t think that quite through…I don’t take risks, but I become less cautious sometimes…you’re more likely to try things” (Charlie, Post-Training) [Q15] “If I fall, what can I do? Like I have to take the challenge…if I don’t challenge myself I cannot move forward. I can fall anytime, that is still there all the time, but since I did the study…like I am more vulnerable to get falls? That I don’t think so, no.” (Ruth, Post-Training)
Category 2b Willingness to try new activities	[Q16] “…when you’re on the walker you can cheat very easily because it’s there. But if you’re on walking sticks…your legs better be doing something and you better have a good balance, good cadence, because the sticks are going to help you a little bit but they’re not going to support you. You have to have the body working to support you.” (Charlie, 8-Weeks Post-Training) [Q17] “…it may not be that I’m necessarily more or less confident, I’m more knowledgeable…what I thought 6 months ago I could do, I realize that I can’t now. And it’s not because I’m less confident, it’s because my expectations I think are more realistic…one of the questions is how confident do you feel that you can walk from the front door to the car. Well I’d never done it before; I was always in a wheelchair. But since I started walking more…not that I’ve tried it, I’m not that confident…I think I’m being realistic; I don’t think that I’m saying to myself you can’t do that therefore you’re not going to try.” (Sharon, 8-Weeks Post-Training)
Quotes for Theme 3: Motivation to keep going	
Category 3a Ability to continue	[Q18] “…I’m a little nervous that maybe I’m not, you know at least here I come 2–3 times a week…trying to get better…once I stop coming here…I don’t have too much motivation to do, and it is not possible to do at home, to do those things, so I’m looking forward to, if I can, get another chance to do another study.” (Ruth, Post-Training)
Category 3b Factors driving motivation	[Q19] “…I don’t push myself to hurt myself, but if it’s hard, what gets me going is I think about that it’s doing me good and I look at the progress I have made…some things now that I do without even thinking, they were was hard as it was when I was in the FES. So, it just keeps me going.” (Sharon, 8-Weeks Post-Training) [Q20] “…I did a lot of that activity before, but sometimes the complacency comes in and that’s when you get a program like this and all of a sudden things are waking up again and you have a feeling, ‘Hey, let’s go again…’ I think I’m motivated, but it’s pretty easy to say, ‘Well, I think I’ll stay home,’ but when new things happen, away you go.” (Charlie, 8-Weeks Post-Training) [Q21] “To stand a little better, firm, so that I can walk better, while standing a little more time than before…I am looking forward to be getting independent by myself, to do my regular life chores, which I am still far, far back from my goal, but I am forwarding, that much I should say, but I still have to go a long way.” (Ruth, Post-Training)

### Theme 1: Perceived benefits across ICF levels

Following the completion of FES + VFBT, participants reported physical improvements including increased muscle strength and endurance, greater body awareness and control, and improved sensory functioning. Psychological improvements, including a feeling of pride and happiness, increased confidence, and a reduced fear of falling, were also reported following the training. These improvements led to functional benefits in activities and participation as participants reported feeling more comfortable moving about their homes as they performed activities of daily living and engaged in social activities.

#### 1a. Impact on impairment

Following the completion of the training program, participants reported a multitude of perceived improvements in body structures and function, such as increased range of motion and muscular strength, and a reduction in muscle spasms and neuropathic pain. Charlie felt that his ankle dorsiflexion may have increased immediately post-training, but was then more convinced that he had improved his range of motion at eight-weeks post-training. Charlie believed that the electrical stimulation *“helped some kind of neural pathway”* because his muscles were more active and he had greater control over them [Q1, see Table 3].

Participants reported that they were able to stand with fewer signs of weakness in their legs. Charlie commented that prior to his participation in FES + VFBT, sometimes his *“leg would just give out.”* However, at the eight-weeks post-training interview he claimed that it was not happening anymore. Ruth thought that she was able to *“stand for a little more without the wiggling.”* Prior to starting the intervention, Carol was hoping to be stronger and to *“trust [her] legs”* more. By the end of the program, immediately post-training, she felt she had achieved *“80% of [her] goals”* and mentioned that her doctor had commented that her legs were getting stronger during a recent appointment [Q2]. Carol also indicated that she had experienced fewer spasms and neuropathic pain episodes [Q2], claiming that since the start of her participation in FES + VFBT the episodes of neuropathic pain happened *“one time, but for a few minutes and it was gone.”*

One unexpected benefit was the perceived improvement in sensory functioning experienced by Charlie in his feet and lower limb muscles [Q3]. While these changes were small, Charlie emphasized that to have *“a sense of feeling down there”* was important and provided him with a feeling of accomplishment [Q4].

#### 1b. Impact on activity

Following completion of FES + VFBT, individuals reported numerous perceived improvements with respect to activity, particularly within their homes [Q5]. Cooking, cleaning, self-dressing, and washroom use/toileting were all activities where participants felt that they had noticeably improved, in large part due to their increased confidence and ability to stand [Q6]. Sharon commented at the eight-weeks post-training interview that her standing endurance had increased as evidenced by her ability to stand with her walker out on the balcony for longer periods of time [Q7]. Ruth noticed immediately post-training that when she is *“doing something on the stove, [she] can stand a little bit more than before.”* Carol commented at the post-training interview that she didn’t like how *“before [she] used to sit on [her] walker and do the chopping.”* Now she reminds herself that *“the walker is only when [she goes] long distances, but it’s not for standing for 10–15 min.”*

Suzanne felt that her confidence was increasing because now she could retrieve items from the floor or reach and grasp something on a shelf. She explained how when using the broom to clean her floor she will *“always put the walker, brake it and sweep a little bit, then move with the walker and sweep.”* Carol reported increased confidence in using the washroom at night. Before participating in FES + VFBT she used to wait a few seconds before walking with her walker to the washroom. Now, she is able to *“get up, bring [her] walker, and…stand up and go to the washroom”* without needing to wait or think about what she is doing.

Several participants noted increased independence in performing activities in daily life. Ruth mentioned how she used to *“call [her] daughter to do those things in [her] room”* but now thinks she is capable of doing them herself. Suzanne echoed those sentiments, commenting that she is no longer *“waiting for other people to come and help [her]”* and emphasized that she would like to either maintain that level of independence or continue to progress further.

Improvements in walking were also noted by several participants. Charlie commented that his wife has *“noticed [him] walking a lot straighter”* around their home. He also believed that he was continuing to increase his stamina since he was able to walk for longer distances. Suzanne reported that she felt increased *“confidence to walk around the house.”* Ruth felt that her mobility had improved [Q8], but admitted *“once [she gets] into the chair [she doesn’t] feel like [getting] up and [doing] something.”*

#### 1c. Impact on participation

Charlie indicated at the eight-weeks post-training interview that he had been to the beach twice with his family and that he was going golfing in a couple of weeks, which were activities he had not been doing prior to FES + VFBT [Q9]. During her time in the intervention, Ruth found herself having to use the washroom at the Lyndhurst Centre, which helped increase her confidence that she can go out in the community for several hours and be able to use an accessible washroom, if necessary. Suzanne explained at the post-training interview that she *“went to the mall and went to the bank”* using only her walker [Q10]. Carol commented that her friend has a large van, which required the use of a walker and a stool to step into the vehicle. However, she explained at the eight-weeks post-training interview how after FES + VFBT she no longer needed the walker to use the stool and told her friend *“to take the walker, [and] put it in the trunk”* when he came to pick her up [Q11].

#### 1d. Inter-related across ICF

On several occasions participants described perceived benefits across more than one ICF level due to the inter-related nature of body structures and function, activity and participation. Charlie felt a benefit of FES + VFBT was feeling increased *“confidence in overall body awareness [and] overall body control.”* Charlie explained that *“because [he’s] balanced it’s easier to keep [his] weight off the walker”* as he can now activate his leg muscles [Q12]. When Charlie visits his son he finds he is able to *“go up and down the stairs like easy”* explaining that he knows *“where [his] feet are and they’re activated.”* When visiting his daughter, Charlie used to go from the car to the front door in his wheelchair, but now uses only a walker.

Ruth felt that her standing balance had improved following FES + VFBT enabling her to try household activities and she noticed that she *“can perform a bit, not too much, but a little bit better than before.”* As she felt herself getting a little more balanced she felt confident enough to move more freely around her home. Ruth commented that *“inside [she feels] happiness.”* Similarly, Carol noticed that she found herself standing more in the kitchen. She explained how before she *“used to stand, but the walker used to be behind [her].”* Carol commented that now she forgets about the walker since her legs are stronger [Q13].

### Theme 2: Change in perceived fall risk & confidence

#### 2a. Risk of falling

Participants reported that their perceived risk of falling stayed the same or was slightly minimized [Q14]. Charlie was able to prevent a fall from happening when he slipped during a vehicle transfer. He explained how *“both of [his] legs shot out and caught [him].”* Likewise, both Sharon and Suzanne believed that their FES + VFBT participation has decreased their risk of falling. Ruth remarked that for her, the risk of falling was always present, but recognized the need to continually challenge herself in order keep moving forward [Q15].

#### 2b. Willingness to try new activities

Individuals indicated that they felt more confident in their physical abilities and that the translation of physical improvements into functional improvements helped to strengthen their feeling of confidence and willingness to try new activities. Sharon described that one day* “[she] sat down on [her] toilet and realized [she] hadn’t put the PT rail down and [she] didn’t go crashing down.”* Charlie described visiting his son’s house for dinner when his son was away. Usually when entering the home, his son is his “*safety net because there one step going from his landing into his house…and there’s nothing for [him] to hold onto except for [his] son.”* However, after completing the FES + VFBT program, Charlie was able to enter the home independently. He had also started using walking sticks rather than a walker [Q16] in some situations, although he was *“not taking a lot of steps…maybe six steps forward, but good steps, balanced steps; there’s no danger.”*

However, several individuals cautioned against feeling over-confident and trying activities that would put them at greater risk of experiencing a fall [Q17]. As Sharon explained, *“as [she gains] confidence [she tries] more things, but then [she] also [is] afraid sometimes that [she is] going to be overconfident and fall; there’s a balance.”* Similarly. Ruth admitted that still wants *“to take [her] power chair everywhere.”* She commented that she is *“not still there to start [her] life with all the [walkers], so that is a big drawback.”*

### Theme 3: Motivation to keep going

#### 3a. Ability to continue

Several participants expressed a desire to continue with the program as they felt that their improvements had not plateaued and that they would continue to benefit from additional training sessions. Several participants also indicated that they were considering continuing using FES as part of their home routine. Charlie mentioned how he had spoken with his physiotherapist about continuing with FES. He explained how he *“found that if [he doesn’t] maintain a routine, it’s easy to go back…so when [he doesn’t] work [his] legs, there’s a difference right away.”* Others commented that they would appreciate the opportunity to participate in future research studies as they had limited options, either privately or within the community, to continue with their recovery [Q18].

#### 3b. Factors driving motivation

Participants reported several factors contributing to their motivation to continue to improve their balance and mobility [Q19]. One such factor was observing physical changes in their bodies [Q20]. Following completion of the training program, Charlie found himself using his more affected limb more *“now because it’s sort of got a new life.”*

The desire to achieve their rehabilitation goals also provided ongoing motivation [Q21]. Sharon was hoping her participation in FES + VFBT would help her to achieve more endurance, strength, stability, balance and mobility. While she felt she did increase, she admitted, *“it’s not finished, it’s an ongoing project.”* Similarly, while Ruth felt that she had improved, *“[she is] still not confident…to go to the grocery store and pick up something, especially milk and juice and heavy things.”* At the beginning of the program, Carol did not think the program would help her, but by the end of the training she wished *“[she] could go back.”* Carol believed that with continued exercise and therapy, she may be able to walk without a gait aid in a year. She mentioned she has a trainer who comes and does stretching and thinks that *“between these training sessions, between her, between [her] exercise, [she feels] that [she is] still progressing.”*

Participating in research was important to several of the participants and was another factor that motivated one to continue to improve balance and mobility. Charlie expressed his desire to continue participating in future studies explaining that he doesn’t *“know what people are going to find in research,”* but he appreciates any improvements in his mobility. Likewise, Suzanne recognized the importance of participating in research mentioning to her friend that even if *“this program is [a] benefit or not, if they study, some other patient, [in the] future, they will get benefit.”*

## Discussion

The experiences of five individuals with chronic motor iSCI who participated in a novel FES + VFBT intervention are described here. This study provides insights into the perceived benefits associated with participating in FES + VFBT, the impact of participation on perceived fall risk and confidence, and reasons for continuing with rehabilitation interventions after program completion. Participants reported a positive impact at all levels of the ICF (e.g. impairment, activity, and participation) following FES + VFBT, suggesting that the perceived benefits of the intervention resulted in a meaningful impact on their lives.

The perceived benefits of FES + VFBT were similar to those reported by Singh et al., [16] who found that improved strength and endurance from a personalized adapted locomotor training (PALT) program contributed to greater independence in activities of daily living for individuals with sub-acute iSCI. Likewise, participants in the PALT program reported increased knowledge about their bodies, improved mood, greater confidence and an increased sense of control [16]. However, individuals enrolled in PALT received, on average, six times more training sessions (range: 49–131 total sessions) than those in FES + VFBT. Training sessions for PALT were also administered four times per week and were 90 min in length [16]. Participants in FES + VFBT indicated that they would have preferred more than 12 training sessions following completion of the intervention and indicated their desire to participate in future studies in order to continue progressing in their recovery.

Our findings highlight the importance of including qualitative inquiry during the development of rehabilitation interventions. In our quantitative evaluation of the FES + VFBT intervention [13], we reported large increases in maximal COP excursion area during the limits of stability test indicating improved dynamic stability. This was supported by functional improvements in the performance of daily activities involving cooking, cleaning and reaching tasks as reported by our participants in their interviews. In contrast, our quantitative study showed little effect (i.e. only 2 participants with improvements > 2 standard deviations) of FES + VFBT on balance confidence, according to the ABC scale [13]. Yet, in the semi-structured interviews all participants expressed feeling more confident in their balance abilities and some felt that they were at less risk of falling following the completion of FES + VFBT. It is possible that the tasks queried on the ABC scale may not reflect the tasks that the participants reported feeling more confident performing in their own homes. Therefore, it is possible that relying solely on the use of quantitative measures may be insufficient to fully capture the experiences of some individuals participating in intervention studies.

Although the inclusion of qualitative methods in clinical trials is rare, there has been an increasing interest among researchers and regulatory bodies to use interviews in addition to traditional, standardized outcomes in interventional studies [21]. The inclusion of qualitative inquiry allows for the evaluation of the meaningfulness of an intervention [14] and of whether or not the participants’ expectations of the intervention were met [22]. Therefore, qualitative approaches can complement the information gained through more traditional performance measures (e.g. Berg Balance Scale) or patient-reported outcome questionnaires (e.g. ABC Scale) by providing an understanding of what and/or how the quantitative scores were achieved. Furthermore, through qualitative methods the outcomes most important to end-users can be identified, directing outcome measure selection for future research [22]. Based on the findings of this qualitative study the quantitative measures used to evaluate the effectiveness of FES + VFBT in future trials should be refined. To allow for better evaluation of the impact of this intervention, clinical assessments that are able to measure the perceived benefits of our participants for activities that are specific and meaningful to them, as reported in their interviews, should be incorporated. For example, our findings may suggest that a customized ABC Scale, which enables participants to select their own meaningful activities for the rating of balance confidence, would benefit future clinical trials investigating balance interventions.

## Limitations

There are several limitations to this study, which warrant consideration. First, our study consisted of a small sample of participants with iSCI that were recruited using purposeful sampling. Therefore, data saturation may not have been reached, the findings may not reflect the larger iSCI population, and researcher and recruitment bias may have occurred [23]. Moreover, the participants were a heterogeneous sample, especially with respect to time post-injury (i.e. ranged from about one year to eight years post-injury). However, since motor and sensory recovery plateau by one year post-injury [24], we believe the variability in injury chronicity likely had little impact on the study findings. Second, some interviews were completed over the phone, which prevented the observation of non-verbal cues during the interview. Non-verbal cues are useful for qualitative analysis as they can help prevent misunderstandings [25] and encourage engagement [26] between the interviewee and interviewer during conversation. Third, interview texts were analyzed using conventional content analysis, which limits interpretation due to its descriptive nature [17].

## Conclusions

Participation in the FES + VFBT program resulted in perceived physical and physiological benefits leading to improvements in daily life. Individuals expressed a desire to continue with the training program as they felt they still had the capacity to improve. Risk of falling was perceived as slightly reduced or unchanged, but participants felt that their balance confidence had increased. However, individuals were wary of over-confidence placing them in situations where they would be more susceptible to a fall. Individuals reported a positive and enjoyable experience, and while benefits differed between participants, each valued their participation in the study. These findings support the continued development of the FES + VFBT intervention as individuals perceived the benefits of participation to be meaningful.

## Data Availability

The datasets used and/or analyzed during the current study are available from the corresponding author on reasonable request.

## Abbreviations

ABC Scale: Activities-specific Balance Confidence Scale
AIS: American Spinal Injury Association Impairment Scale
AP: Anterior–posterior
BBS: Berg Balance Scale
COP: Centre-of-pressure
FES: Functional electrical stimulation
FES + VFBT: Functional electrical stimulation plus visual feedback balance training
ICF: International Classification of Functioning, Disability and Health
iSCI: Incomplete spinal cord injury
Mini-BESTest: Mini-Balance Evaluation Systems Test
PALT: Personalized adapted locomotor training
SCI: Spinal cord injury
VFBT: Visual feedback balance training
